# Supplementary material for: Examination of optical coherence tomography findings in patients with pregabalin use disorder
Source: PeerJ. 2024 Nov 11;12:e18395. doi: 10.7717/peerj.18395 (PMC11562773; doi:10.7717/peerj.18395)
Supplement: Supplemental Information 1 [file peerj-12-18395-s001.docx]

| job status | sex | marital status | education status | smoking | ADDITIONAL PSYCHIATRIC DIAGNOSIS |
| --- | --- | --- | --- | --- | --- |
| 1- yes | 1. male | 1 married | 1- LITERATE | 1- yes | 0- none |
| 2- no | 2- female | 2- single | 2- PRIMARY SCHOOL | 2- no | 1- personality disorder |
|  |  | 3- widow/divorced | 3- MIDDLE SCHOOL |  | 2- Psychotic disorders |
|  |  |  | 4- HIGH SCHOOL |  | 3-Depression |
|  |  |  | 5- UNIVERSITY |  | 4- Bipolar disorder |
|  |  |  | 6- UNI + |  | 5- Anxiety disorders |
